# Significance and value of non-traded ecosystem services on farmland

**DOI:** 10.7717/peerj.762

**Published:** 2015-02-17

**Authors:** Harpinder Sandhu, Steve Wratten, Robert Costanza, Jules Pretty, John R. Porter, John Reganold

**Affiliations:** 1 School of the Environment, Flinders University, Adelaide, SA, Australia; 2 Bio-Protection Research Centre, Lincoln University, Lincoln, New Zealand; 3 Crawford School of Public Policy, The Australian National University, Canberra, ACT, Australia; 4 School of Biological Sciences and Essex Sustainability Institute, University of Essex, Colchester, UK; 5 Faculty of Science, University of Copenhagen, Taastrup, Denmark; 6 Natural Resources Institute, University of Greenwich, London, UK; 7 Department of Crop and Soil Sciences, Washington State University, Pullman, WA, USA

**Keywords:** Ecosystem services, Arable farmland, Agroecosystems, Economic value, New Zealand, Externalities

## Abstract

**Background.** Ecosystem services (ES) generated within agricultural landscapes, including field boundaries, are vital for the sustainable supply of food and fibre. However, the value of ES in agriculture has not been quantified experimentally and then extrapolated globally.

**Methods.** We quantified the economic value of two key but contrasting ES (biological control of pests and nitrogen mineralisation) provided by non-traded non-crop species in ten organic and ten conventional arable fields in New Zealand using field experiments. The arable crops grown, same for each organic and conventional pair, were peas (*Pisum sativum*), beans (*Phaseolus vulgaris*), barley (*Hordeum vulgare*), and wheat (*Triticum aestivum*). Organic systems were chosen as comparators not because they are the only forms of sustainable agriculture, but because they are subject to easily understood standards.

**Results.** We found that organic farming systems depended on fewer external inputs and produced outputs of energy and crop dry matter generally less than but sometimes similar to those of their conventional counterparts. The economic values of the two selected ES were greater for the organic systems in all four crops, ranging from US$ 68–200 ha^−1^ yr^−1^ for biological control of pests and from US$ 110–425 ha^−1^yr^−1^ for N mineralisation in the organic systems versus US$ 0 ha^−1^yr^−1^ for biological control of pests and from US$ 60–244 ha^−1^yr^−1^ for N mineralisation in the conventional systems. The total economic value (including market and non-market components) was significantly greater in organic systems, ranging from US$ 1750–4536 ha^−1^yr^−1^, with US$ 1585–2560 ha^−1^yr^−1^ in the conventional systems. The non-market component of the economic value in organic fields was also significantly higher than those in conventional fields.

**Discussion.** To illustrate the potential magnitude of these two ES to temperate farming systems and agricultural landscapes elsewhere, we then extrapolate these experimentally derived figures to the global temperate cropping area of the same arable crops. We found that the extrapolated net value of the these two services provided by non-traded species could exceed the combined current global costs of pesticide and fertiliser inputs, even if utilised on only 10% of the global arable area. This approach strengthens the case for ES–rich agricultural systems, provided by non-traded species to global agriculture.

## Introduction

Agroecosystems consume non-marketed ecosystem services (ES) provided by non-traded species (e.g., predatory beetles and parasitic wasps provide biological control of insect pests, soil biota provide nutrient cycling) for the production of marketed ES (Porter et al., 2009; Wratten et al., 2013). These take the form of traded plant and animal products, which comprise the economic returns derived from farmland (Matson et al., 1997; Pretty et al., 2006). To meet the increasing food demand of a growing human population and their changing consumption patterns (Pretty, 2013), current farming technology based on high inputs is expected to result in a four-fold increase in nitrogen fertiliser and pesticide use by 2050 (Tilman et al., 2001). Currently, large amounts of fertiliser and pesticide are used inefficiently (Vitousek et al., 2009) and half the nitrogen circulating in ecosystems globally is already anthropogenic (Vitousek et al., 1997). The key challenge is to enhance productivity of agroecosystems in a sustainable manner by reducing external costs and increasing the understanding and enhancement of farmland ES. Meeting this challenge has been called ‘sustainable intensification’ (Pretty, Toulmin & Williams, 2011; Garnett et al., 2013; Pretty & Bharucha, 2014). These problems are being increasingly stated and analysed but, to improve the contribution of ES to sustainable production of food and fibre, there is an increasing need to recognise the total economic value of non-marketed ES (Bayon & Jenkins, 2010) and then identify practices and policies to enhance them (Schutter, 2010). Sustainable intensification will require that many non-renewable resources (e.g., fossil fuel-based pesticides and fertilizers) are at least partially replaced by renewable resources, such as ES based on biological control or nitrogen fixation.

Agroecosystems cover c. 5 billion hectares worldwide and include c. 1.5 billion ha arable land. Arable land comprises non-pasture area under annual crops, such as cereals, legumes, and oilseed crops. Globally, agriculture offers a significant opportunity to increase global ES by developing land management practices that favour ES provision (Sandhu, Crossman & Smith, 2012; Sandhu & Wratten, 2013). Agriculture can be considered to be the largest ecological experiment on Earth, with a high potential to damage global ES but also to enhance them via ecologically informed design of agroecosystems that deliver and value marketed and non-marketed ES (Porter et al., 2009). The Millennium Ecosystem Assessment (MEA, 2005) completed by science and policy communities provided a new framework for analysing socio-ecological processes and suggested that agriculture may be the largest threat to biodiversity and ecosystem function of any single human activity. Similarly, the MEA raised awareness of ecosystems and their services but the global environment continues to degrade because of a lack of a coherent plan of action (MEA, 2005). Recently, the United Nations has established the Intergovernmental Science-Policy Platform on Biodiversity and Ecosystem Services (IPBES, 2010) to translate ecosystem science into action, and to track the drivers and consequences of ecosystem change worldwide. This action plan is focused on strengthening assessment, relevant policy and associated science at a range of spatial and temporal scales. A potential new paradigm shift at global level indicates a move towards a ‘green economy’ as discussed at the Rio + 20 Summit (United Nations Environment Program, 2010; United Nations, 2012). Providing food to 9 billion people worldwide by 2050 will need greater global coherence to develop agricultural systems that utilise and maintain high levels of ES so that they can provide sustainable economic well-being and food security within ecological and financial constraints (Pretty et al., 2006; The Royal Society, 2009; Schutter, 2010).

The large increase in global grain production during the past 50 years was due mainly to high-yielding varieties and breeds, mechanisation, irrigation, and large increases in the use of pesticides and fertilisers (Tilman et al., 2001). However, a number of developing countries have not seen a per capita increase in food production, due to limited availability of these costly systems and inputs (Vitousek et al., 2009). In addition, some of the environmental consequences of the ‘Green Revolution’ and related intensifications have been high (Hazell & Wood, 2008).

This study seeks to address these global problems by exploring the potential economic value of two key ES in agriculture—the biological control of insect pests by soil-surface predators and the mineralisation of plant nutrients (nitrogen in this case). These two ES were chosen because (i) they have been substituted globally, largely by pesticides and fertilisers, respectively, and (ii) data on pesticide and fertiliser use are widely available for most global cropping areas. It follows that any substitution for agrochemical inputs by these and many other ES on farmland could reduce input costs and provide direct benefits to farmers (lower variable costs) and the environment (reduced external costs). For pests, this approach can also reduce the risk of pesticide resistance developing in pests.

In this study, we first assess the above two key ES using field experiments in ten conventional and ten organic fields in New Zealand (Sandhu et al., 2008), and then calculate the effect of organic and conventional practices on the delivery of the two key ES from arable fields under four crop types: peas, beans, barley and wheat. We chose conventional and organic systems for comparisons not because organics are the only form of agricultural systems that could be described as sustainable (Pretty & Bharucha, 2014), but because standards and protocols exist for organic cultivation of these crops. We conclude by discussing the potential relative magnitude of these ES in temperate arable areas in 110 countries in 15 global regions (see Table S1) as one plausible scenario. We also provide an economic and environmental justification for enhancing the use of ES in agriculture for increasing production and ensuring food and ecological security.

## Materials and Methods

### Field assessment of ES in New Zealand arable farmland

Field trials were conducted from 2004–2006 in 20 arable fields spread across the province of Canterbury, the main arable area of New Zealand, comprising 125,000 ha of arable land (Statistics NewZealand, 2006). Canterbury’s climate is strongly influenced by warm north-westerly winds and receives an average annual rainfall of 500–800 mm in its lowland agricultural areas. Soils are developed mainly from wind-blown volcanic dust (loess). They are generally lightly structured and of medium fertility for agricultural purposes.

The experimental design consisted of two arable farming systems (organic and conventional), both including annual grain crops such as cereals, oilseeds and legumes. Although many management practices exist between ‘organic’ and ‘conventional,’ these systems were chosen as they differ markedly in terms of synthetic fertiliser and pesticide use. There were about 25 organic and 500 conventional arable farms in Canterbury at the time of study (MAF, 2006). A list of arable farmers in Canterbury was obtained from the Foundation for Arable Research (www.far.org.nz), Lincoln, New Zealand, and OPENZ (Organic Products Exporters of New Zealand; www.organicsnewzealand.org.nz) provided the contacts for all organic farmers. The latter were contacted first by sending a letter, followed by a telephone call and a meeting to collect detailed information about the farming practices, such as crop rotations and the crops grown, as well as soil type. Ten organic fields were selected from the above totals, one field being used per farm based on there being an arable crop grown at the time of the survey. Some organic farms were not growing arable crops so they were not selected for this study. Subsequently, conventional arable farmers within 5 km of the selected organic farms were contacted. These were selected within this radius because they were growing similar crops on similar soil types. These farmers were practising high-input intensive mixed farming, which included arable crops. Each field pair consisted of two fields, one organic and one conventional, and although not directly adjacent to each other, fields chosen in each pair had the same microclimate, soil type and crop type and rotation (Reganold et al., 1993; Drinkwater et al., 1995). The 10 organic/conventional field pairs had the following crops: two organic/conventional field pairs growing peas (*Pisum sativum*), two field pairs with beans (*Phaseolus vulgaris*), three field pairs in barley (*Hordeum vulgare*) and three more in wheat (*Triticum aestivum*). The mean field area of organic fields was 10.3 ha (range 8–14 ha) and conventional fields was 10.4 ha (range 7–15 ha).

### Organic arable farming

Organic agriculture is a production system that aims to optimise the ‘health’ of soil, plants, animals and people (Reganold et al., 2001). It virtually excludes synthetic fertilizers and pesticides, emphasizing instead building up the soil with composts and animal and green manures, managing pests using ES, rotating and diversifying crops and livestock, and enhancing functional biodiversity (Wachter & Reganold, 2014). This system claims to produce adequate high-quality yields in an environmentally, economically and socially sustainable way (Wachter & Reganold, 2014). New Zealand has approximately 40,000 ha of certified organic farmland with exports of US$ 50 million per annum. The main products are kiwifruit (5% of that sector), pip fruit (10% of the pip fruit sector, including apples at 5%), process vegetables and arable crops, the latter being 2% of that sector. Organic farming that meets certified organic standards is being practised on 36 million ha worldwide by 1.8 million farmers across 162 countries, with a value of c. US$ 63 billion (Willer, Lernoud & Kilcher, 2013).

### Conventional arable farming

We define conventional farming as systems with high rates of synthetic inputs, such as pesticides and fertilisers, to control pests, maintain soil fertility and produce maximum outputs per ha (Wachter & Reganold, 2014). ‘Pesticides’ include insecticides, herbicides, fungicides and others such as growth regulators. Costs of these products are reported in terms of active ingredient and they do not include the other components of the final formulation (FAOSTAT, 2014). This intensive system is able to produce large amounts of food and raw materials to meet an increasing demand. In New Zealand, MAF categorises organic and conventional area separately, there are 424,000 ha under conventional arable agriculture (MAF, 2006). Globally, there are 1.54 billion arable ha (FAOSTAT, 2014).

### Biological control of pests

Biological control is an ES provided by predators, parasitoids and bacteria and fungi. The component of this service which is delivered by soil-surface invertebrate predators (one of many natural enemy guilds) was assessed experimentally in this work and data were then used to estimate the value of this guild in the selected fields. Biological control usually contributes more to organic agriculture than it does to conventional farming as the former is more dependent on such services to keep pest and other populations low.

Here, the predation rates on aphids and fly eggs were assessed using field experiments (Sandhu et al., 2008). Aphids and larvae of root-feeding fly pests are important in many arable and other crops in Canterbury and elsewhere (Van Emden & Harrington, 2007). Live pea aphids (*Acyrthosiphon pisum* Harris) as well as frozen eggs of the blowfly (*Calliphora vicina* R.D.) were used. The latter simulated those of fly pests which lay eggs on or near the soil surface. Predation rates on these two prey types were assessed on two dates in all 20 study fields in November 2004 and January 2005. The aphid densities were selected in November 2004 (1/25 cm^2^ and 4/25 cm^2^) and January 2005 (4/25 cm^2^ and 10/25 cm^2^) based on previous studies in arable land (Winder, 1990; Ekbom, Wiktelius & Chiverton, 1992; Winder et al., 1994). Two densities of blowfly eggs were used based on the literature on the abundance of carrot rust fly egg populations (Burn, 1982; Burn, 1984). Published egg densities are in the range of 3–8/25 cm^2^ in the field (Burn, 1982). Predation rate was assessed using ‘prey surrogates’ comprising 25 cm^2^ water-proof sandpaper squares pinned to the soil surface by wooden toothpicks (Merfield, Wratten & Navntoft, 2004; Frank et al., 2007). Live aphids (dorsal side uppermost) were glued onto the sandpaper (P150, Norton) using 3M repositionable glue in a grid pattern with 1 cm between aphids. The blowfly eggs were not glued onto the surface but were placed in a similar pattern. The sandpaper sheets were pinned at the field boundary, the field centre and midway between the two in two transects (5 m apart) in each field and had a 225 cm^2^ metal plate supported 10 cm above to protect them from rain.

Predation rate was calculated as the average of number and proportion of ‘prey’ types removed over 24 h periods during the two study periods. At each site, each type of ‘prey’ at both densities (minimum and maximum) was positioned 1 m apart at the locations described above. For each prey type for each period, overall mean prey disappearance was calculated separately from the means of the two prey densities. Predation rate (%removal/24 h) was required per field to calculate the economic value of biological control of aphids and carrot rust fly. Therefore, no further analysis was conducted on the effect of season or field position on predation rates.

The economic value of this ‘background’ (i.e., unmanipulated) biological control of aphids and fly eggs was estimated by using avoided cost (AC) of pesticides (insecticides only), based on New Zealand prices (for the (insecticides , labour and fuel) and total avoided cost (TAC) of pesticides (insecticides only), which included US$ 61.00 ha^−1^ yr^−1^ as their external costs (Pretty et al., 2000). UK data were used for the latter, as appropriate data are not available in New Zealand. The mean costs of insecticides used on Canterbury arable farms to manage aphids and root pests are US$ 35.00 and US$ 30.00 ha^−1^ application^−1^, respectively. A further US$ 10.50 ha^−1^ is spent as an application cost (labour and fuel) for each pest on each occasion (Sandhu et al., 2008).

The economic estimates of the value of ‘background’ predation presented here are based on an ‘instantaneous’ (24 h) assessment of a complex predation process, while economic results based on AC and TAC are provided on a ha^−1^ yr^−1^ basis. Conventional farmers should use pesticides only to reduce pest populations below economic thresholds. It is assumed in this study that when the instantaneous reduction of pest numbers by soil-surface predators over 24 h reduces the population below the economic threshold level, then this is equivalent to one effective pesticide application. Without the availability of a predator–prey model to estimate the decrease in pest populations following a 24 h predation event, the estimates of the economic value (AC and TAC) of biological control are based on the assumption that conventional farmers apply two such applications per year. This is reasonable based on current spray recommendations (Chapman, 2004).

To calculate the economic value of aphid predation, three densities (with equal probability of occurrence) were used as follows: density 1 (d1; 10 aphids/25 cm^2^, maximum density used in the predation work), density 2 (d2; 7.5 aphids/25 cm^2^) and density 3 (d3; 6.25 aphids/25 cm^2^). Densities 2 and 3 are between the economic threshold and the maximum density used in the predation work. The economic threshold was based on the work by Thies, Roschewitz & Tscharntke (2004). These authors gave an economic threshold for 3–5 aphids per shoot for *Sitobion avenae, Metopolophium dirhodum and Rhopalosiphum padi* in wheat fields. This is converted here to a unit-area measure, giving an economic threshold of five aphids/25 cm^2^ based on numbers of shoots per unit area (McCloy, 2004). For each of the three densities (d1, d2 and d3), the number of aphids consumed by the soil-surface predators (based on predation rate in that field) were estimated for the two periods during November 2004 and January 2005.

For the carrot rust fly, an economic threshold based on egg densities is not available; this is not surprising, as assessing these densities is technically very demanding (Burn, 1984). Therefore, three economic thresholds (ET1; 6.25 eggs/25 cm^2^, ET2; 5 eggs/25 cm^2^, ET3; 3.75 eggs/25 cm^2^) within the published densities used in this predation work with equal probability of occurrence were simulated. In each field, predation rates were used to estimate the decrease in pest populations below the simulated economic thresholds.

The economic value based on AC and TAC was assigned to the fields in which predation rate was able to bring the pest population below the economic threshold. Then, the values obtained for the two periods (November 2004 and January 2005) were added to provide the total economic value in each field.

### Nitrogen mineralisation

Organic matter comminution and decomposition by invertebrates and microorganisms is one of the most important ES provided by soil (Brady & Weil, 2008). Through decomposition, plant residues are broken down, releasing previously organically bound nutrients such as nitrogen, for use by plants (Edwards & Arancon, 2004). The rate of *N* mineralisation in this work was assessed by using bait lamina probes (Kratz, 1998; Torne, 1990; Jacometti, Wratten & Walter, 2007; Sandhu et al., 2008). Those used here were made of rigid plastic and were 16 cm long, 0.6 cm broad and 1 mm thick, with sixteen 2 mm holes (Kratz, 1998; Edwards & Arancon, 2004). The latter were filled with a gel comprising by weight cellulose (65%), agar-agar (15%), bentonite (10%) and wheat bran (10%) that matches to some extent the key constituents of dead plant material on or in the soil (Edwards & Arancon, 2004). The strips were inserted into the soil at the field boundary, the field centre and midway between the two in two transects (5 m apart) in each field. The probes were left in the ground for 10 days. Soil microorganisms and invertebrates consume the ‘bait’ and the number of holes that are empty (partially or fully) gives a relative measure of the rate of mineralisation (Kratz, 1998; Torne, 1990).

The economic value of plant nutrient mineralisation provided by soil microorganisms and invertebrates was assessed using data on mineralisation of organic matter obtained from the 20 fields as follows:

(1) \begin{eqnarray*} {N}_{\mathrm{min}}=n\times b\times v\times k{10}^{-3}\hspace{0.167em} \mathrm{kg} \end{eqnarray*}

where, *N*_min_ = amount of nitrogen mineralised *n* = total amount of nitrogen (%) in soil *b* = bulk density of soil (g cm^−3^) v = volume of soil (cm^3^) *k* = percentage mineralisation (%)

The percentage of organic matter mineralised in each field was calculated from this by using the nutrient mineralisation rate from the bait lamina probes. Total organic matter content in the fields was estimated using the total weight of soil (obtained from bulk density at 10 cm depth) and total nitrogen obtained from soil testing. It was based on the assumptions that the ratio of organic matter to nitrogen is 20:1 (Brady & Weil, 2008). The amount of organic matter mineralised in each field was calculated from this assumption by using nutrient mineralisation rate from the bait lamina probes. The total amount of nitrogen mineralised was estimated from Eq. (1) and valued at the equivalent price of N kg^−1^ (US$ 0.84kg^−1^; Sandhu et al., 2008) providing the economic value of nutrient mineralisation.

### Total economic value of ecosystem services

This was calculated by adding non-market and market (produce) of ES for each of the 10 organic and conventional fields as follows:

(2) \begin{eqnarray*} \sum {\mathrm{ES}}_{\mathrm{total}}=\sum {\mathrm{ES}}_{\mathrm{non}\text{-}\mathrm{market}}+\sum {\mathrm{ES}}_{\mathrm{market}} \end{eqnarray*}

In this case, ‘non-market ES’ comprise background (natural occurring) biological control of pests and N mineralisation, as they are not traded in the market. Market ES comprise the four crops (peas, beans, barley and wheat) which are traded in market.

### Other data from the two farming systems

Data on pH, bulk density, total nitrogen, total carbon, grain yields, energy inputs and outputs, and monetary inputs and outputs, along with the assessment of the non-market key ES were determined (see Table S2). Data were analysed using SPSS Version 20 and the two farming systems were compared using *t*-tests.

## Results

### Inputs and outputs for organic and conventional arable cropping systems

The organic practices used here depended on fewer external inputs and produced outputs of energy and crop dry matter generally lower than but sometimes similar to those of conventional cropping in New Zealand (Table 1). The reported ranges are within those obtained globally (Seufert, Ramankutty & Foley, 2012). Compared to conventional yields, organic ones were notably lower for barley and wheat but similar for beans and peas (Table 2). Market prices for peas and beans were similar for organic and conventional produce. However, they were higher for organic barley and wheat (Table 2). Lower yields in barley and wheat were compensated by higher market prices so the total value of produce was similar for peas, beans and barley but organic wheat had a higher overall value in market (Table 2).

**Table 1:** The ranges of inputs and outputs for organic and conventional cropping systems in the province of Canterbury, New Zealand.

	Organic agriculture	Conventional agriculture
**Inputs** (**ha**^**−1**^ **yr**^**−1**)^		
Energy (GJ ha^−1^ yr^−1^)	3.3–7.8	5–9.8
Industrial N fertilizer (kg)	–	30–80
Insecticides (kg a.i.)	–	0.9–1.2
Fungicides (kg a.i.)	–	4.3–5.5
Herbicides (kg a.i.)	–	0.2–0.8
Irrigation (mm)	16–30	25–60
**Outputs** (**ha**^**−1**^ **yr**^**−1**^)		
Energy (GJ ha^−1^ yr^−1^)	48–79	48–109
Grain (t dry matter)	4–6.3	4–10.5

**Notes.**

GJ gigajoules a.i. active ingredient t metric tons

**Table 2:** Yield, market price and total value of four crops in 10 organic and 10 conventional fields.

	Yield t ha^−1^		Market price US$ t^−1^	
	Org	Cnv	Org	Cnv
Peas	3.7	4	490	500
Peas	6	5	490	500
Beans	16.7	16.7	165	140
Beans	17.2	16	165	140
Barley	5	8.5	350	175
Barley	4	8.7	350	175
Barley	4.2	9	350	175
Wheat	5	10	700	182
Wheat	5	11	700	182
Wheat	6	7.5	700	182
Mean	7.28	9.64	446	235.1
Min	3.7	4	165	140
Max	17.2	16.7	700	500

**Notes.**

Org organic fields Cnv conventional fields

### Predation rates on aphids and fly eggs

The predation rates of aphids ranged from 20.2–82.0% in organic fields and 0–5.9% in conventional ones (Table 3). Predation rates of blowfly eggs in 24 h ranged from 24.2–76.0% in organic fields and 0–6% in conventional ones (Table 3). These differences were compared using *t*-tests for unequal sample variances (Table 3). Predation rate of aphids was significantly higher in organic fields than in the conventional ones (*p* < 0.001). The same was true for blowfly eggs (*p* < 0.001).

**Table 3:** Predation rates and N mineralisation rates in organic and conventional fields.

	Aphid predation rate (%)		Fly egg predation rate (%)		Mineralisation rate (%)	
	Org	Cnv	Org	Cnv	Org	Cnv
Peas	24	0	25	0	8	10
Peas	20.2	0	26	0	6	3
Beans	42.8	5.9	24.2	3	17	7
Beans	42.8	5.9	24.2	3	17	7
Barley	26	0	26	0	2	4
Barley	42	2.3	40	6	8	8
Barley	36	2.3	36	6	8	8
Wheat	76	0	76	0	8	2
Wheat	82	0	74	3	4	5
Wheat	76	0	76	4.5	4	11

**Notes.**

Org organic fields Cnv conventional fields

The economic value of the biological control of aphids and blowfly eggs combined was in the range of US$ 68.00–200.00 ha^−1^ yr^−1^ (TAC; mean US$ 1,30.00) in 10 organic fields (Table 4). None of the conventional fields had any economic value for biological control at any ‘population’ density, due to extremely low predation rates.

**Table 4:** The economic value of the biological control of aphids and blowfly eggs combined and *N* mineralisation in organic and conventional fields.

	Biological controlTotal avoided cost US$ ha^−1^ yr^−1^		N mineralisationTotal avoided cost US$ ha^−1^ yr^−1^		Total economic value US$ ha^−1^ yr^−1^	
	Org	Cnv	Org	Cnv	Org	Cnv
Peas	102	0	194	244	296	244
Peas	68	0	110	60	178	60
Beans	103	0	425	122	528	122
Beans	103	0	425	122	528	122
Barley	110	0	174	140	284	140
Barley	130	0	220	226	350	226
Barley	120	0	220	220	340	220
Wheat	180	0	220	70	400	70
Wheat	200	0	160	150	360	150
Wheat	180	0	156	220	336	220

**Notes.**

Org organic fields Cnv conventional fields

### Rate of nitrogen mineralisation

The mean rate of mineralisation was calculated as the mean rate of removal of baits (based on complete and incomplete removal and is given in Table 3. The extent of removal in organic fields was 2–17% (mean 8.2%) and 2–11% (mean 6.5%) in conventional ones. There were no significant differences between organic and conventional fields in this case. The range in the economic value of mineralisation rates was from US$ 110.00 to 425.50 ha^−1^ yr^−1^ (mean US$ 230.00 ha^−1^ yr^−1^) in organic fields and US$ 60.00–244.00 ha^−1^ yr^−1^ (mean US$ 157.00 ha^−1^yr^−1^) in conventional ones (Table 4).

### Combined economic value of biological control of pests and N mineralisation

The combined economic values (in 2012 US$) of the two ES were greater (*P* < 0.001) for the organic systems in all four crop types, ranging from US$ 178–528 ha^−1^yr^−1^ and from US$ 60–244 ha^−1^ yr^−1^ in the conventional systems (Table 4). These values are within reported ranges in previous studies in the USA (Pimentel et al., 1997) and at a global scale (Costanza et al., 1997; Costanza et al., 2014), although in those cases value transfer (Wilson & Hoehn, 2006) rather than experimental methods was used.

### Combined economic value of market and non-market ecosystem services

**Figure 1:**
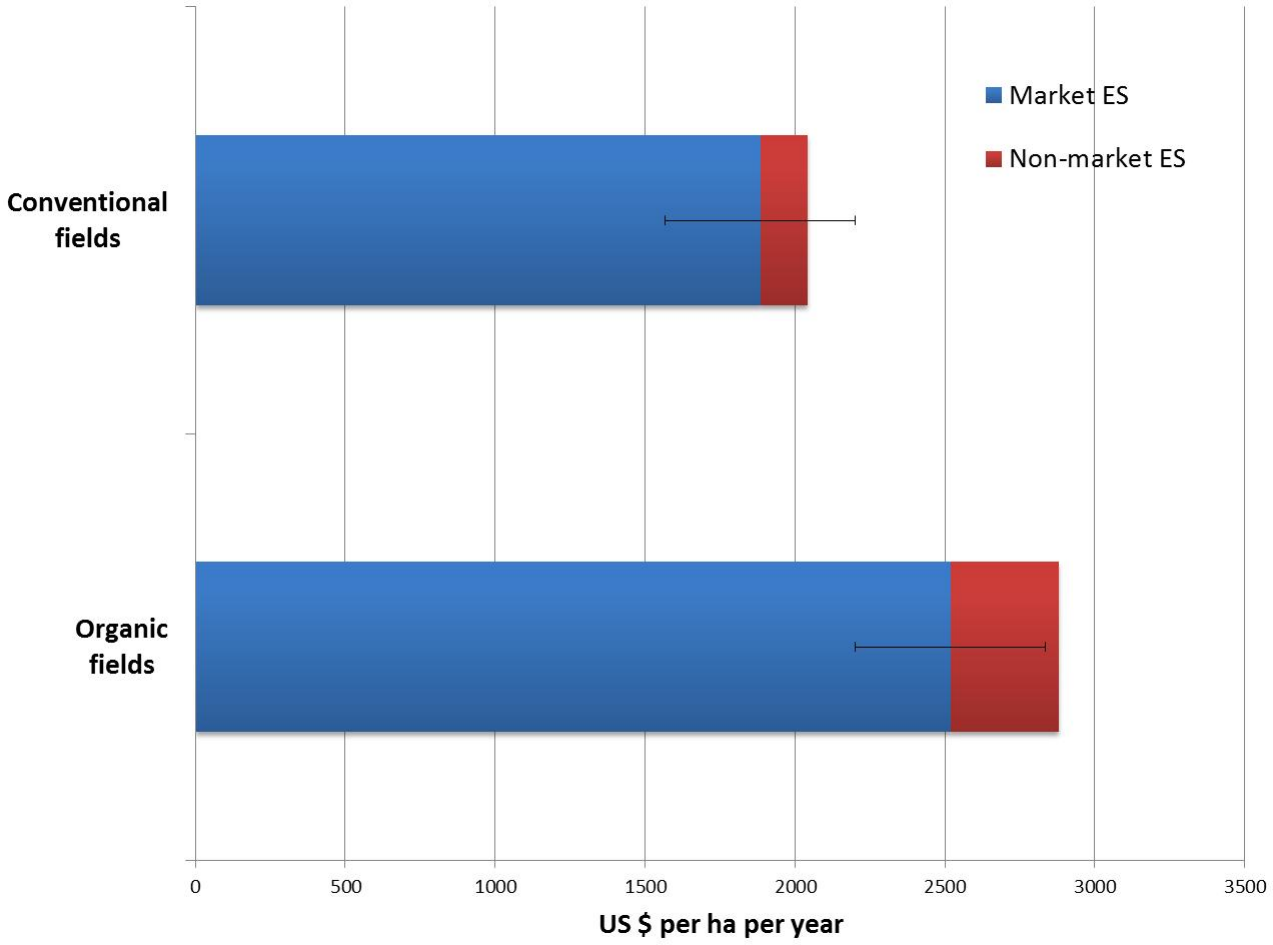
The mean economic value of ES (US$ ha^−1^ year^−1^), including market and non-market ES, in ten organic and ten conventional fields. Error bars are SE for the market ES.

The combined economic value, including market value of the crop and the non-market value of the two other ES ranged from US$ 1750–4536 ha^−1^ yr^−1^ in organic fields and US$1585–2560 ha^−1^ yr^−1^ in conventional fields (*P* = 0.01; Table 5). The mean non-market component of the organic fields was also significantly higher (*P* < 0.001) than that for conventional ones (Fig. 1).

**Table 5:** Total economic value of ecosystem services (market and non-market) in organic and conventional fields.

	Total economic value US$ ha^−1^ yr^−1^	
	Org	Cnv
Peas	2,109	2,244
Peas	2,138	2,560
Beans	3,283	2,460
Beans	3,366	2,362
Barley	2,034	1,627
Barley	1,750	1,746
Barley	1,810	1,795
Wheat	3,900	1,890
Wheat	3,860	2,152
Wheat	4,536	1,585

**Notes.**

Org organic fields Cnv conventional fields

## Discussion

### Role of ES in organic and conventional agriculture

Conventional arable farming consumes large amounts of inputs in terms of pesticides, fertilisers, energy and water as compared to organic farming systems (Table 1, Sandhu et al., 2008). However, there was no significant difference between the total ES outputs from conventional and organic farming systems in this study (Table 2). Conventional arable farming can sometimes suppress the ability of farmland to provide ES, as was evident from the studied conventional fields with no non-market economic value (Table 4; Sandhu et al., 2008; Sandhu, Wratten & Cullen, 2010). However, there is debate on the generally lower yields in organic agriculture worldwide compared with those from conventional farming (Seufert, Ramankutty & Foley, 2012; De Ponti, Rijk & Van Ittersum, 2012). Many research-driven agro-ecological practices can increase conventional and organic yields (Pretty, Toulmin & Williams, 2011). Here, we show that high-input costs can at least be partially reclaimed by converting conventional arable land to forms of agriculture with high ES delivery (Table 3). Also, currently used high-yielding varieties tend to have to be supported by high inputs of fertilisers, pesticides and water. Pesticide and fertiliser use may be associated with risks such as pest resistance and outbreaks and overdose of nutrients, which add to the increasing cost of production to farmers (MEA, 2005). In contrast, many research-led agro-ecological approaches have produced protocols that are available for deployment now (Schutter, 2010; Wratten et al., 2013). From our field research, it was demonstrated that the two non-marketed ES investigated here have high economic value in organic fields (Table 4; Sandhu et al., 2008). They can also be enhanced by using the principles of SPUs/‘ecological engineering’ (Gurr, Wratten & Altieri, 2004).

Incorporating an ES approach into production landscapes does not require widespread conversion to organic agriculture. Rather, it requires an understanding of the need to achieve uptake and continuation of agri-environment schemes based on sound ecological knowledge and business decisions, so that they can effectively protect biodiversity and enhance farmland ES in many agricultural sectors. New mechanisms such as payments or rewards for ecosystem services (PES) (Pagiola, Bishop & Landell-Mills, 2002; Wunder, 2005) and greater recognition of the barriers to uptake and continuation of agri-innovations are required to ensure the widespread deployment of ES-enhancement strategies to maintain and enhance agricultural sustainability without compromising yield (UNCTAD, 2008; The Royal Society, 2009).

### Economic and environmental justification for enhancing the use of ES in agriculture

Increasing concerns about food supply and security will require a wide range of sustainable agricultural practices to fulfill the food demand of a growing population and its changing consumption patterns (Ericksen, Ingram & Liverman, 2009; Godfray et al., 2010; Glover et al., 2010). A key future challenge is to improve the understanding of ES and environmental consequences of agricultural intensification, so that they can be managed and mitigated, respectively, to achieve food security.

To illustrate the potential of the relative magnitudes of these two ES for world farming, we extrapolate, with appropriate caveats, these values for the two non-market ES obtained from the New Zealand farming systems studied here to temperate arable areas in 110 countries in 15 global regions (see Table S1) along with the economics of total *N* consumed and total pesticide use in those regions (in 2012 US $). The 15 regions were selected on the basis of temperate climatic conditions occurring in up to two-thirds of the total country area, as the field data used here were derived from New Zealand temperate conditions (see Table S1). Information on the area under four previously-selected crop types, their production and the amount of fertiliser and pesticide used for each of the 110 countries in the 15 regions were obtained from FAOSTAT (2014).

The potential economic value of the two ES (biological control value for organic fields only, it was zero in conventional fields) and N mineralisation value for conventional and organic fields) was extrapolated from the New Zealand arable study (see Table S3, Sandhu et al., 2008; Sandhu, Wratten & Cullen, 2010). See Table S3, so three, not four, separate extrapolations were made (see column 6, 10 and 11 in Table S4).

**Figure 2:**
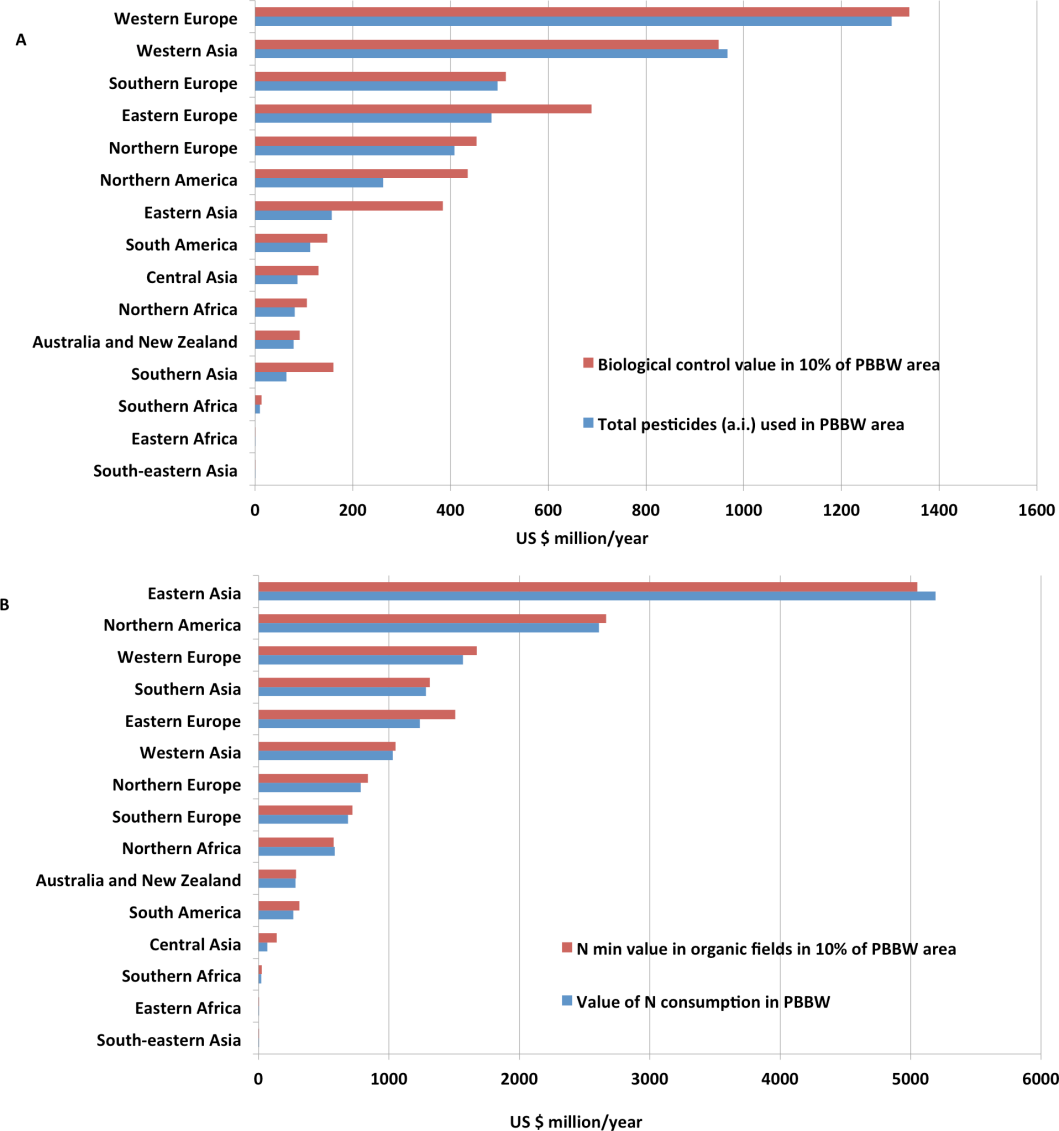
(A) Economic value of biological control ES in 10% of PBBW (peas, beans, barley and wheat) area compared with the total pesticides value in 15 global regions. (B) Economic value of nitrogen mineralisation ES (N min) in 10% of PBBW (peas, beans, barley and wheat) area compared with the value of nitrogen consumption in 15 global regions.

This extrapolation resulted in very high potential economic values of ES (see Table S4) for an organic scenario. This was more than the total direct costs (not including external costs) of pesticides (insecticides only) and fertilisers in these 15 regions, even if only 10% of the global arable area was converted to systems of higher ES delivery, such as organic (Table 6). For the one component of biological control measured here (i.e., predation by one soil-surface predatory guild), its value from organic fields is 3 times that of the current pesticide input in conventional arable cropping globally (see columns 5 and 6 in Table S4). If only 10% of the global arable area is converted to methods of sustainable intensification, including organic agriculture, then the total value of biological control still exceeds the costs of current pesticide use in conventional agriculture (see columns 5 and 7 in Table S4; Fig. 2A).

**Table 6:** Total value of inputs in 15 global regions for target crops (PBBW = peas, beans, barley and wheat) and economic value of two key ecosystem services combined for 100% and 10% of the global arable area under organic management for above crops.

	Regions	Total value of pesticides and fertilisers in PBBW area (US$ million yr^−1^)	Total value based on two ES in PBBW area (US$ million yr^−1^)	Total value based on two ES in 10% of PBBW area (US$ million yr^−1^)
1	Eastern Africa	0.3	0.8	0.3
2	Northern Africa	665.9	836.1	682.9
3	Southern Africa	28.9	115.7	37.6
4	South America	381.5	1,165.7	459.9
5	Northern America	2,872.4	5,139.6	3,099.1
6	Central Asia	154.1	1,323.8	271.0
7	Eastern Asia	5,347.6	6,225.8	5,435.4
8	Southern Asia	1,347.2	2,615.0	1,474.0
9	South-eastern Asia	0.02	3.1	0.3
10	Western Asia	1,994.6	2,026.5	1,997.9
11	Eastern Europe	1,720.8	6,487.5	2,197.5
12	Northern Europe	1,192.5	2,191.4	1,292.4
13	Southern Europe	1,180.4	1,731.2	1,235.4
14	Western Europe	2,871.8	4,286.4	3,013.2
15	Australia and New Zealand	360.5	531.8	377.7
	Total	20,119.1	34,680.9	21,575.3

Extrapolating the rate of background N mineralisation from conventional fields indicates a decrease in potential economic value in seven regions if that rate is applied to them; compare columns 9 and 10 in Table S4. For the organic fields studied here, there is a decrease in the potential economic value of N mineralisation in only two regions when New Zealand N mineralisation rates are extrapolated (see columns 9 and 11 in Table S4). However, the total value of this ES in organic fields exceeds the total costs of fertiliser if 10% of the global arable area is converted to organic or similar appropriate low-input agriculture (see columns 9 and 12 in Table S4; Fig. 2B).

The extrapolations used here are only illustrations of the potential relative magnitudes of ES in conventional and organic fields and are not precise forecasts. This approach, however, can help improve the understanding of the potential contribution of ES provided by non-traded species to global agriculture. It does not advocate large-scale conversion to organic practices. However, if only 10% of the global arable area utilised such ES-enhancing techniques, then this study shows that the total ES value can then surpass the total cost of inputs (Table 6).

The two key ES evaluated here, which are provided by non-traded species, have the potential to deliver pest population reductions and vital nutrients. In contrast, the global ecological and economic consequences of reduced biodiversity and the ES it provides are substantial (Balvanera et al., 2006; Bayon & Jenkins, 2010; Chapinet al., 2000). Similarly, the benefits to agriculture of key ES are substantial as demonstrated here; in most cases the organisms which deliver these ES are not traded in markets; i.e., they have a value but no price. Enhancing these and other ES in agriculture has the potential to reduce its ecological footprint (Wackernagel & Rees, 1996). Although some of the regions in this study are already responding to agri-environment schemes and demands for market-based instruments for pesticide-free food (Soil Association, 2010), in other regions, this can be addressed by applying the above instruments as well as other ‘payment for ecosystem services’ (PES) schemes (FAO, 2007). This will help farmers in these regions and others to be compensated for any loss of incomes due to the possible slight decline in production by adopting sustainable agricultural practices in the short to medium term. Finding these and other ways to minimise the opportunity costs involved in enhancing farmland biodiversity is ‘one of the most important scientific, social and political challenges of the near future’ (Tylianakis, 2013). In the long term, ES enhancement will help to optimise production and sustainability of farms and will benefit a suite of ES, not only the two examined here (Wratten et al., 2012). Importantly, however, and perhaps surprisingly, ‘developed’ regions with histories of extensive agricultural research can all benefit greatly by adopting ES-intensive farming to the extent that the economic costs of nitrogen and insecticide use can be equalled or surpassed if only 10% of their farmland adopted the two ES-intensive practices studied here. Importantly, this potential is much greater, as many ES can be enhanced on farmland (Wratten et al., 2013). For the fifteen global regions examined here, and on the same basis as above, the two key ES studied here exceed US$ 20 billion ha^−1^ yr^−1^ (Table 6).

### Caveats in extrapolations to global level

Extrapolation of the economic value data from New Zealand case study to global agriculture is provided here to explore ‘what if’ scenario, in order to contribute towards the current debate on sustainable intensification of agriculture and associated environmental impacts (Tilman et al., 2011). Economic values obtained in this extrapolation should be used with caution to further explore the role of ES to global agriculture. We also provide caveats of the methods used in this study.

1. The methods used in this study to assess two field-based ES present a ‘snapshot’ in time, but as more and better information becomes available, better estimates of the total value of these ES and how they may change with time can be obtained.
2. It was assumed that the background data for the value of these two ES in New Zealand arable land were similar to the equivalent values in the other world regions analysed. This is justified by the fact that New Zealand agriculture has many components that make it adequately representative for the purpose of this estimate. It builds on European, North American, and Australian agriculture in terms of cropping patterns, crop rotations, resource use and inputs. Global pesticide and fertiliser use in temperate areas falls within the ranges found in New Zealand agriculture (FAOSTAT, 2014). Also, the global extrapolations are comparable to earlier non-experimental studies that extrapolated data using value-transfer methods (Wilson & Hoehn, 2006). The latter value transfer approaches also have limitations based on the sources of data and the extrapolations involved (Lindhjem & Navrud, 2008). In the current work, data on ES are collected at the focal study sites, not extrapolated from the literature. However, even if the latter are wrong by an order of magnitude, the potential economic value of farmland ES demonstrated here is much higher than is recognised in national and international farming policies.
3. We also recognise that the values obtained here ignore the on-farm costs of using the ES as substitutes for purchased inputs, and the costs of off-farm externalities (negative effects on human health and the environment) of mineral fertilizers and synthetic pesticides (Pretty et al., 2000). Farmers use purchased inputs instead of ES because they believe that the capability of the latter to substitute for those inputs in most cases is limited. Even when ES are enhanced, this comes at a cost in terms of the inputs involved (e.g., labour, organic material). Therefore, the limitation of the approach used here, the exclusion of on-farm costs of using ES, overestimates the value of ES, whereas the exclusion of off-farm externalities results in an underestimate. It is beyond the scope of this research to estimate the magnitude of these two factors. Moreover, if there was a large-scale reduction in the use of oil-derived inputs, then the market prices of inputs would decline. Using the prices at current levels of demand for agrochemical inputs, therefore, may overstate the values attributed to their reduction. However, prices of oil and its derivatives are unlikely to decline markedly in the near future (Economist, 2013). If they do environmental harm, through the externalities of farming, this is likely to increase the need for enhanced delivery of ES.
4. Regional climatic effects, land-use and crop management changes and their costs, rate of uptake by farmers, and several other factors are also not accounted for in these calculations.
5. We did not examine the effect on yields under organic scenario if two ES are used under 10% conversion area.
6. We assumed that the yield of each crop is dependent on the background value of each of the two ES. Therefore, to calculate the value of each of the two ES under two regimes for four crops in different countries, ratios of value of ES to crop yield were derived from the New Zealand study (Table S3). This ratio was used to estimate the economic value of each of the two ES (for four crops in each country) in the 15 global regions.

## Conclusion

Conventional farming often suppresses the delivery of non-marketed ES whereas organic and other benign agricultural practices enhance it. Organic agriculture recognises the high economic value of non-marketed ES in production of marketed ES (grains, in this study). Such a benefit-cost ratio offers significant returns to farmers and renewable, cost-effective alternatives to fossil-fuel based inputs in many agroecosystems, not limited to organic agriculture. Improving the ES-richness of agriculture requires a considerably higher uptake of agroecological approaches which make economic sense to farmers as well as protecting the biodiversity which enhances farmland ES (Schutter, 2010; Hodson et al., 2010; Tylianakis, 2013). This study strengthens the case for more diversified, ES–rich, integrated agricultural systems that enhance functional agricultural biodiversity, avoid expensive inputs, minimise external costs and are less energy intensive. Part of the currently-available agricultural technical knowledge and efforts can be diverted for the further development and extension of sustainable intensification of agricultural practices and protect the livelihood of millions of farmers.

## Supplemental Information

10.7717/peerj.762/supp-1 Supplemental Information Supplemental Information 1

10.7717/peerj.762/supp-2 Table S1 The regions and countries used in this analysis, total arable area, area under four crops (PBBW; peas, beans, barley and wheat) and their levels of production. Million hectare (M Ha), Million Tonnes (M Tonnes).

10.7717/peerj.762/supp-3 Table S2 Cropping history, pH, bulk density, total *C* and *N* in 10 organic (Org) and 10 conventional (Cnv) fields.

10.7717/peerj.762/supp-4 Table S3 The mean economic value of biological control of pests and *N* mineralisation, yield and ratio of ES to yield under conventional and organic arable fields in New Zealand.

10.7717/peerj.762/supp-5 Table S4 Total temperate arable land, inputs, outputs, economic value of target crops (PBBW; peas, beans, barley and wheat), and economic value of two key ecosystem services in 15 global regions (see text for details): Biological control value for organic fields only (zero value in conventional fields) and nitrogen mineralisation (N min) value for conventional (Cnv) and organic (Org) fields. Million hectare (M Ha), Million Tonnes (M Tonnes).
