# Supplementary material for: Significance and value of non-traded ecosystem services on farmland
Source: PeerJ. 2015 Feb 17;3:e762. doi: 10.7717/peerj.762 (PMC4338771; doi:10.7717/peerj.762)
Supplement: Supplemental Information [file peerj-03-762-s001.docx]

**Supplementary Information**

**Significance and value of non-traded ecosystem services on farmland**

Harpinder Sandhu*^1^, Steve Wratten^2^, Robert Costanza^3^, Jules Pretty^4^,

John R. Porter^5^, John Reganold^6^

**Calculation of the potential economic value of the two ES**

We assumed that the yield of each crop is dependent on the background value of each of the two ES. Therefore, to calculate the value of each of the two ES under two regimes for four crops in different countries, ratios of value of ES to crop yield were derived from the New Zealand study (Table S3) (Equation S1). This ratio and Equation S2 (below) were used to estimate the economic value of each of the two ES (for four crops in each country) in the 15 global regions (Equation S3); see below.

$R_{ES F}= V_{ES F C}/Y_{C}$ Equation S1

$V_{ES F C}= R_{ES F} X A_{Reg C}X Y_{Reg C}$ Equation S2

Total economic value of these two arable ES for each global region in US $ yr^-1^:

$V_{ES Reg F C}= \sum{ES}_{F C}$ Equation S3

Where,

R = the ratio of the economic value of ES to yield ($ t^-1^ yr^-1^)

V = economic value ($ yr^-1^)

ES = ecosystem service (biological control of pests or N mineralisation)

F = field type (conventional or organic)

A = area (hectare)

Y = yield (tonnes)

C = crop type (peas, beans, barley or wheat)

Reg = region

Economic values provided in the Table S4 do not overestimate the value of two ES during extrapolation. Table S4 provides total temperate arable land (column 2) in 15 regions with area (column 3) and production (column 4) figures of four crops (PBBW; peas, beans, barley and wheat) considered in this study. Column 5 is about the value of pesticides (insecticides only) in 15 regions. Column 6 estimates the biological control values for each region based on Equation S3 with ratios obtained from Table S3. Column 7 provides the total value of biological control when 10% area uses the values for biological control values obtained from PBBW area. Column 7 values are not 10% of the column 6. These values are not for the 10% area only but for the total area under PBBW crops. These are the total value that can be attributed to the total PBBW area including, 10% of the value used from Biological control values and rest 90% is the avoided costs of pesticides (i.e., 90% of the total pesticide values). Therefore in some cases the total value is more than the value attributed to the column 6. See Figure 2A for the economic value of biological control in 10% of PBBW area compared with the total pesticides used in 15 global regions. Column 8 is the value of nitrogen used in PBBW area with its economic value in column 9. Column 10 estimates the nitrogen mineralisation value from conventional fields data and column 11 uses the organic fields data from New Zealand case study (Table S3 and equation S3). Column 12 estimates if 10 % area uses nitrogen mineralisation value obtained from organic fields data. It is not 10% of the value provided in column 11. See Figure 2B for the economic value of nitrogen mineralisation ES (N min) in 10% of PBBW (peas, beans, barley and wheat) area compared with the value of nitrogen consumption in 15 global regions. These calculations also demonstrate that we are not over estimating the economic value of two ES for global agriculture.

**TABLE LEGENDS**

Table S1 The regions and countries used in this analysis, total arable area, area under four crops (PBBW; peas, beans, barley and wheat) and their levels of production. Million hectare (M Ha), Million Tonnes (M Tonnes).

Table S2 Cropping history, pH, bulk density, total C and N in 10 organic (Org) and 10 conventional (Cnv) fields.

Table S3 The mean economic value of biological control of pests and N mineralisation, yield and ratio of ES to yield under conventional and organic arable fields in New Zealand.

Table S4 Total temperate arable land, inputs, outputs, economic value of target crops (PBBW; peas, beans, barley and wheat), and economic value of two key ecosystem services in 15 global regions (see text for details): Biological control value for organic fields only (zero value in conventional fields) and nitrogen mineralisation (N min) value for conventional (Cnv) and organic (Org) fields. Million hectare (M Ha), Million Tonnes (M Tonnes).
