# Supplementary material for: Significance and value of non-traded ecosystem services on farmland
Source: PeerJ. 2015 Feb 17;3:e762. doi: 10.7717/peerj.762 (PMC4338771; doi:10.7717/peerj.762)
Supplement: Table S1 [file peerj-03-762-s002.docx]

Table S1

|  |  |  | **Total arable area**  **(M Ha)** | **Area - Peas, Beans, Wheat and Barley**  **(M Ha)** | **Production PBBW**  **(M Tonnes)** |
| --- | --- | --- | --- | --- | --- |
| 1 | Eastern Africa | Eriteria | 0.6 | 0.06 | 0.01 |
| 2 |  | Mayotte | 0 | 0 | 0 |
| 3 | Northern Africa | Algeria | 7.46 | 1.92 | 3.51 |
| 4 |  | Egypt | 3.01 | 1.26 | 7.61 |
| 5 |  | Libya | 1.75 | 0.33 | 0.21 |
| 6 |  | Morocco | 8.06 | 4.61 | 2.37 |
| 7 |  | Tunisia | 2.75 | 1.21 | 1.98 |
| 8 |  | Western Sahara | 0 | 0.02 | 0 |
| 9 | Southern Africa | Lesotho | 0.30 | 0.06 | 0 |
| 10 |  | South Africa | 14.50 | 0.75 | 2.16 |
| 11 |  | Swaziland | 0.17 | 0 | 0 |
| 12 | South America | Argentina | 32.50 | 6.54 | 18.34 |
| 13 |  | Chile | 1.29 | 0.25 | 1.17 |
| 14 |  | Malvinas | 0 |  |  |
| 15 |  | Guyana | 0.42 | 0 | 0 |
| 16 |  | Paraguay | 4.30 | 0.41 | 0.87 |
| 17 |  | Peru | 3.70 | 0.40 | 0.48 |
| 18 |  | Uruguay | 1.35 | 0.39 | 1.01 |
| 19 | Northern America | Canada | 45.10 | 14.22 | 34.24 |
| 20 |  | Greenland | 0 | 0 | 0 |
| 21 |  | Saint pierre | 0 | 0 | 0 |
| 22 |  | USA | 170.42 | 22.98 | 62.26 |
| 23 | Central Asia | Kazakhstan | 22.70 | 18.95 | 14.55 |
| 24 |  | Kyrgystan | 1.28 | 1.00 | 0.52 |
| 25 |  | Tajikstan | 0.71 | 0.32 | 0.72 |
| 26 |  | Turkemenistan | 1.85 | 0.91 | 2.76 |
| 27 |  | Uzbekistan | 4.30 | 1.43 | 6.28 |
| 28 | Eastern Asia | China | 140.63 | 26.49 | 114.85 |
| 29 |  | Korea | 4.39 | 0.52 | 0.75 |
| 30 |  | Japan | 4.32 | 0.30 | 1.19 |
| 31 |  | Mongolia | 0.85 | 0.11 | 0.11 |
| 32 | Southern Asia | Afghanistan | 8.53 | 2.70 | 4.85 |
| 33 |  | Bangladesh | 7.97 | 0.46 | 0.78 |
| 34 |  | Bhutan | 0.12 | 0.01 | 0.01 |
| 35 |  | Iran | 16.86 | 5.86 | 18.22 |
| 36 |  | Nepal | 2.35 | 0.77 | 1.57 |
| 37 |  | Pakistan | 21.50 | 9.07 | 23.65 |
| 38 | South-eastern Asia | Cambodia | 3.80 | 0.06 | 0.05 |
| 39 |  | Timor-leste | 0.17 | 0 | 0 |
| 40 | Western Asia | Armenia | 0.40 | 0.16 | 0.43 |
| 41 |  | Azerbaijan | 1.85 | 0.70 | 1.79 |
| 42 |  | Bahrain | 0 | 0 | 0 |
| 43 |  | Cyprus | 0.11 | 0.03 | 0.06 |
| 44 |  | Georgia | 0.46 | 0.07 | 0.12 |
| 45 |  | Iraq | 5.20 | 3.30 | 2.9 |
| 46 |  | Israel | 0.30 | 0.09 | 0.16 |
| 47 |  | Jordan | 0.14 | 0.06 | 0.03 |
| 48 |  | Kuwait | 0.01 | 0 | 0 |
| 49 |  | Lebanon | 0.14 | 0.06 | 0.15 |
| 50 |  | Palestine | 0.10 | 0.03 | 0.05 |
| 51 |  | Qatar | 0.01 | 0 | 0.05 |
| 52 |  | Saudi Arabia | 3.40 | 0.47 | 2.6 |
| 53 |  | Syria | 4.73 | 3.03 | 4.83 |
| 54 |  | Turkey | 21.92 | 11.52 | 24.69 |
| 55 |  | UAE | 0.07 | 0 | 0 |
| 56 | Eastern Europe | Belarus | 5.53 | 1.14 | 3.42 |
| 57 |  | Bulgaria | 3.08 | 1.28 | 2.82 |
| 58 |  | Czech Republic | 3.03 | 1.33 | 5.88 |
| 59 |  | Hungary | 4.59 | 1.45 | 5.08 |
| 60 |  | Poland | 12.50 | 3.37 | 12.39 |
| 61 |  | Moldova | 1.82 | 0.47 | 0.53 |
| 62 |  | Romania | 8.55 | 2.28 | 3.61 |
| 63 |  | Russia | 121.57 | 32.49 | 65.79 |
| 64 |  | Slovakia | 1.37 | 0.58 | 2.05 |
| 65 |  | Ukraine | 32.43 | 10.30 | 20.21 |
| 66 | Northern Europe | Aland island | 0 | 0 | 0 |
| 67 |  | Channel island | 3.40 | 0 | 0 |
| 68 |  | Denmark | 2.30 | 1.32 | 7.63 |
| 69 |  | Estonia | 0.59 | 0.22 | 0.71 |
| 70 |  | Faeroe islands | 0 | 0 | 0 |
| 71 |  | Finland | 2.25 | 0.74 | 2.79 |
| 72 |  | Guernsey | 0 | 0 | 0 |
| 73 |  | Iceland | 0 | 0 | 0 |
| 74 |  | Ireland | 1.06 | 0.25 | 3.36 |
| 75 |  | Isle of Man | 0 | 0 | 0 |
| 76 |  | Jersey | 0 | 0 | 0 |
| 77 |  | Latvia | 1.18 | 0.37 | 1.16 |
| 78 |  | Lithuania | 1.83 | 0.75 | 2.43 |
| 79 |  | Norway | 0.85 | 0.23 | 0.88 |
| 80 |  | Svalbard and Jan mayen islands | 0 | 0 | 0 |
| 81 |  | Sweden | 2.64 | 0.69 | 3.73 |
| 82 |  | UK | 6.08 | 2.76 | 18.43 |
| 83 | Southern Europe | Albania | 0.57 | 0.08 | 0.27 |
| 84 |  | Andorra | 0 | 0 | 0 |
| 85 |  | Bosnia nd Herzegovina | 1.02 | 0.10 | 0.33 |
| 86 |  | Croatia | 0.85 | 0.23 | 1.04 |
| 87 |  | Gibraltar | 0 | 0 | 0 |
| 88 |  | Greece | 2.54 | 0.86 | 1.95 |
| 89 |  | Holy see | 0 | 0 | 0 |
| 90 |  | Italy | 7.17 | 2.46 | 8.44 |
| 91 |  | Malta | 0 | 0 | 0.01 |
| 92 |  | Montenegro | 0.17 | 0 | 0 |
| 93 |  | Portugal | 1.08 | 0.10 | 0.21 |
| 94 |  | San Marinao | 0 | 0 | 0 |
| 95 |  | Serbia | 3.29 | 0.67 | 1.90 |
| 96 |  | Slovenia | 0.17 | 0.05 | 0.20 |
| 97 |  | Spain | 12.70 | 5.20 | 18.12 |
| 98 |  | Yugoslav and Macedonia | 0 | 0 | 0 |
| 99 | Western Europe | Austria | 1.38 | 0.51 | 2.26 |
| 100 |  | Belgium | 0.84 | 0.24 | 1.85 |
| 101 |  | France | 18.43 | 7.10 | 42.83 |
| 102 |  | Germany | 11.87 | 4.97 | 31.38 |
| 103 |  | Liechtenstein | 0 | 0 | 0 |
| 104 |  | Luxemborg | 0.06 | 0.02 | 0.11 |
| 105 |  | Monaco | 0 | 0 | 0 |
| 106 |  | Netherlands | 1.05 | 0.18 | 1.25 |
| 107 |  | Switzerland | 0.40 | 0.13 | 0.77 |
| 108 | Australia and New Zealand | Australia | 22.00 | 8.54 | 9.62 |
| 109 |  | New Zealand | 0.50 | 0.04 | 0.36 |
| 110 |  | Norfolk island | 0 | 0 | 0 |
|  | **Total** |  | **881.98** | **237.82** | **651.28** |
