# Supplementary material for: Significance and value of non-traded ecosystem services on farmland
Source: PeerJ. 2015 Feb 17;3:e762. doi: 10.7717/peerj.762 (PMC4338771; doi:10.7717/peerj.762)
Supplement: Table S2 [file peerj-03-762-s003.docx]

Table S2

|  | **Cropping history** | | |  |  |  |  |
| --- | --- | --- | --- | --- | --- | --- | --- |
| **Field type** | **2002-03** | **2003-04** | **2004-05** | **pH** | **Volume weight**  **(g ml^-1^)** | **Total carbon (%)** | **Total nitrogen (%)** |
| Org | Pasture | Clover | Peas | 5.8 | 1.02 | 2.5 | 0.25 |
| Org | Pasture | Wheat | Peas | 6 | 0.97 | 2.8 | 0.27 |
| Org | Pasture | Wheat | Beans | 5.8 | 0.93 | 3.2 | 0.32 |
| Org | Pasture | Wheat | Beans | 5.7 | 0.95 | 3.1 | 0.34 |
| Org | Pasture | Linseed | Barley | 6.2 | 1 | 2.4 | 0.21 |
| Org | Beans | Pasture | Barley | 6.2 | 1.03 | 2.6 | 0.25 |
| Org | Beans | Pasture | Barley | 6.1 | 1.1 | 2.7 | 0.25 |
| Org | Wheat | Rape | Wheat | 6.2 | 0.95 | 3.4 | 0.34 |
| Org | Beans | Pasture | Wheat | 6 | 1 | 2.6 | 0.26 |
| Org | Linseed | Peas | Wheat | 6.3 | 1.01 | 2.4 | 0.23 |
| Cnv | Barley | Wheat | Peas | 6.3 | 0.92 | 3 | 0.31 |
| Cnv | Barley | Wheat | Peas | 5.7 | 0.94 | 3.1 | 0.3 |
| Cnv | Wheat | Pasture | Beans | 5.5 | 1.08 | 2.1 | 0.2 |
| Cnv | Wheat | Pasture | Beans | 5.6 | 1.06 | 2.1 | 0.25 |
| Cnv | Pasture | Wheat | Barley | 6 | 0.98 | 2.8 | 0.27 |
| Cnv | Rape | Wheat | Barley | 6.4 | 1.02 | 3 | 0.29 |
| Cnv | Rape | Wheat | Barley | 6.2 | 1.02 | 2.9 | 0.3 |
| Cnv | Pasture | Barley | Wheat | 6.2 | 0.96 | 3 | 0.3 |
| Cnv | Barley | Pasture | Wheat | 5.7 | 0.96 | 3 | 0.28 |
| Cnv | Barley | Pasture | Wheat | 6 | 0.98 | 2.5 | 0.24 |
