# Supplementary material for: Significance and value of non-traded ecosystem services on farmland
Source: PeerJ. 2015 Feb 17;3:e762. doi: 10.7717/peerj.762 (PMC4338771; doi:10.7717/peerj.762)
Supplement: Table S3 [file peerj-03-762-s004.docx]

Table S3

|  | Beans | Barley | Peas | Wheat |
| --- | --- | --- | --- | --- |
| **Conventional fields** |  |  |  |  |
| Yield t ha^-1^ | 16.7 | 8.6 | 4.3 | 9.5 |
| Biological control value US$ ha^-1^ yr^-1^ | 0 | 0 | 0 | 0 |
| N mineralisation value US$ ha^-1^ yr^-1^ | 122 | 196 | 153 | 147 |
| Ratio of Biological control value to yield US $t^-1^yr^-1^ | 0 | 0 | 0 | 0 |
| Ratio of N mineralisation value to yield US $ t^-1^ yr^-1^ | 7.3 | 23 | 35 | 15 |
|  |  |  |  |  |
| **Organic fields** |  |  |  |  |
| Yield tha^-1^ | 16.7 | 4.5 | 4.9 | 5.5 |
| Biological control value US$ ha^-1^ yr^-1^ | 103 | 120 | 85 | 188 |
| N mineralisation value US$ ha^-1^ yr^-1^ | 425 | 204 | 152 | 160 |
| Ratio of Biological control value to yield US $ t^-1^ yr^-1^ | 6 | 26 | 17 | 34 |
| Ratio of N mineralisation value to yield US $ t^-1^ yr^-1^ | 25 | 45 | 31 | 29 |
