# Supplementary material for: Significance and value of non-traded ecosystem services on farmland
Source: PeerJ. 2015 Feb 17;3:e762. doi: 10.7717/peerj.762 (PMC4338771; doi:10.7717/peerj.762)
Supplement: Table S4 [file peerj-03-762-s005.docx]

Table S4

|  | **1** | **2** | **3** | **4** | **5** | **6** | **7** | **8** | **9** | **10** | **11** | **12** |
| --- | --- | --- | --- | --- | --- | --- | --- | --- | --- | --- | --- | --- |
|  | **Regions** | **Total arable area**  **(M Ha)** | **Area - PBBW**  **(M Ha)** | **Production of PBBW**  **(M Tonnes)** | **Total pesticides (a.i.) used in PBBW area**  **(US$ million yr^-1^)** | **Biological control value in PBBW area**  **(US$ million yr^-1^)** | **Biological control value in 10% of PBBW area**  **(US$ million yr^-1^)** | **N used in PBBW area**  **(M Tonnes)** | **Value of N consumption in PBBW**  **(US$ million**  **yr^-1^)** | **N min value in Cnv fields PBBW area**  **(US$ million yr^-1^)** | **N min value in Org fields in PBBW area**  **(US$ million**  **yr^-1^)** | **N min value in Org fields in 10% of PBBW area**  **(US$ million yr^-1^)** |
| 1 | Eastern Africa | 0.6 | 0.07 | 0.01 | 0.2 | 0.3 | 0.2 | 0.01 | 0.06 | 0.3 | 0.5 | 0.1 |
| 2 | Northern Africa | 23.0 | 9.4 | 15.7 | 81.0 | 325.8 | 105.4 | 0.6 | 584.9 | 315.2 | 510.3 | 577.4 |
| 3 | Southern Africa | 14.9 | 0.8 | 2.1 | 9.6 | 45.1 | 13.2 | 0.02 | 19.2 | 43.6 | 70.6 | 24.4 |
| 4 | South America | 43.5 | 8.0 | 21.8 | 113.2 | 454.2 | 147.3 | 0.3 | 268.3 | 439.4 | 711.4 | 312.6 |
| 5 | Northern America | 215.5 | 37.2 | 96.5 | 261.6 | 2002.7 | 435.7 | 3.1 | 2610.8 | 1937.6 | 3136.8 | 2663.4 |
| 6 | Central Asia | 30.8 | 22.6 | 24.8 | 87.0 | 515.8 | 129.9 | 0.07 | 67.0 | 499.0 | 807.9 | 141.1 |
| 7 | Eastern Asia | 150.2 | 27.4 | 116.9 | 157.4 | 2426.0 | 384.2 | 6.1 | 5190.2 | 2347.1 | 3799.8 | 5051.1 |
| 8 | Southern Asia | 57.3 | 18.8 | 49.1 | 64.2 | 1019.0 | 159.7 | 1.5 | 1282.9 | 985.8 | 1596.0 | 1314.2 |
| 9 | South-eastern Asia | 3.9 | 0.07 | 0.06 | 0.01 | 1.2 | 0.1 | 0.01 | 0.01 | 1.1 | 1.9 | 0.1 |
| 10 | Western Asia | 38.9 | 19.5 | 38.05 | 966.6 | 789.6 | 948.9 | 1.2 | 1028.0 | 764.0 | 1236.8 | 1048.9 |
| 11 | Eastern Europe | 194.5 | 54.7 | 121.8 | 484.3 | 2528.0 | 688.7 | 1.4 | 1236.4 | 2445.7 | 3959.5 | 1508.7 |
| 12 | Northern Europe | 22.2 | 7.3 | 41.1 | 408.5 | 853.9 | 453.0 | 0.9 | 784.0 | 826.1 | 1337.5 | 839.4 |
| 13 | Southern Europe | 29.6 | 9.8 | 32.5 | 495.8 | 674.6 | 513.7 | 0.8 | 684.5 | 652.6 | 1056.6 | 721.7 |
| 14 | Western Europe | 34.0 | 13.1 | 80.4 | 1302.8 | 1670.3 | 1339.5 | 1.8 | 1568.9 | 1615.9 | 2616.1 | 1673.6 |
| 15 | Australia and New Zealand | 22.5 | 8.5 | 9.9 | 78.7 | 207.2 | 91.5 | 0.3 | 281.8 | 200.5 | 324.6 | 286.1 |
|  | Total | 881.9 | 237.8 | 651.2 | 4511.4 | 13514.1 | 5411.7 | 18.5 | 15607.6 | 13074.5 | 21166.8 | 16163.6 |
